# Characterisation of Age-Dependent Beta Cell Dynamics in the Male db/db Mice

**DOI:** 10.1371/journal.pone.0082813

**Published:** 2013-12-06

**Authors:** Louise S. Dalbøge, Dorthe L. C. Almholt, Trine S. R. Neerup, Efstathios Vassiliadis, Niels Vrang, Lars Pedersen, Keld Fosgerau, Jacob Jelsing

**Affiliations:** 1 Department of Histology, Gubra ApS, Hørsholm, Denmark; 2 Department of Research and Development, Zealand Pharma A/S, Glostrup, Denmark; 3 Department of Stereology, Visiopharm, Hørsholm, Denmark; University of Barcelona, Faculty of Biology, Spain

## Abstract

**Aim:**

To characterise changes in pancreatic beta cell mass during the development of diabetes in untreated male C57BLKS/J db/db mice.

**Methods:**

Blood samples were collected from a total of 72 untreated male db/db mice aged 5, 6, 8, 10, 12, 14, 18, 24 and 34 weeks, for measurement of terminal blood glucose, HbA_1c_, plasma insulin, and C-peptide. Pancreata were removed for quantification of beta cell mass, islet numbers as well as proliferation and apoptosis by immunohistochemistry and stereology.

**Results:**

Total pancreatic beta cell mass increased significantly from 2.1 ± 0.3 mg in mice aged 5 weeks to a peak value of 4.84 ± 0.26 mg (P < 0.05) in 12-week-old mice, then gradually decreased to 3.27 ± 0.44 mg in mice aged 34 weeks. Analysis of islets in the 5-, 10-, and 24-week age groups showed increased beta cell proliferation in the 10-week-old animals whereas a low proliferation is seen in older animals. The expansion in beta cell mass was driven by an increase in mean islet mass as the total number of islets was unchanged in the three groups.

**Conclusions/Interpretation:**

The age-dependent beta cell dynamics in male db/db mice has been described from 5-34 weeks of age and at the same time alterations in insulin/glucose homeostasis were assessed. High beta cell proliferation and increased beta cell mass occur in young animals followed by a gradual decline characterised by a low beta cell proliferation in older animals. The expansion of beta cell mass was caused by an increase in mean islet mass and not islet number.

## Introduction

In recent decades obesity and type 2 diabetes (T2D) have raised increasing concern worldwide due to their alarming rise in prevalence [1,2]. Today about 347 million people globally are diabetic [2]. This number is estimated to increase to about 439 million by 2030 with the highest rise occurring in developing countries [1,2]. Clinical manifestation of T2D is characterised by insulin resistance, impaired insulin secretion and pancreatic beta cell dysfunction [3-6]. Human studies have consistently indicated that beta cell mass in patients with T2D is decreased compared with healthy individuals [6-8]. Studies in rodents suggest that pancreatic beta cells have the capacity to compensate for an increased metabolic load and insulin demand by increasing the beta cell mass and function in order to maintain normal blood glucose [9-11]. However, when the metabolic demands exceed the compensatory capacity of the increased beta cell mass and insulin secretion, hyperglycaemia and T2D will develop [5,12,13]. 

The homozygous db/db mouse carrying a deleterious point mutation in the leptin receptor gene [14-16] has been extensively used as an experimental model of T2D. Db/db mice are obese, hyperphagic, hypometabolic and develop diabetes at a relatively young age of around 8 weeks. The blood glucose values increase until death at about 8 month of age [17]. Diabetes development in db/db mice strongly resembles that in human T2D as insulin resistance and hyper-insulinemia precede hyperglycemia [17-19]. In db/db mice, plasma insulin concentrations have been reported to peak at about 2-3 months of age followed by a gradual decline [17,20]. The increase in plasma insulin concentrations is believed to be coupled with an increased beta cell mass followed by a gradual decrease in beta cell mass [17,19,21-23]. Despite being broadly used in studies of pancreatic beta cell modulation, the age-related beta cell dynamics in untreated db/db mice have not been investigated in detail [23-26]. However, without a sufficient knowledge of the age-dependent beta cell dynamics in the untreated db/db mouse conclusive interpretation of pharmaceutical compound-induced changes in beta cell mass is difficult. 

The present study was designed to fully characterise and investigate changes in pancreatic beta cell mass during the development of glucose intolerance in male C57BLKS/J db/db mice aged from 5 weeks (when they are considered pre-diabetic) to 10 weeks (early diabetic) to 24 weeks (late-stage diabetic) and even to 34 weeks. The combined use of stereological methods for estimation of beta cell mass along with a full characterisation of changes in glucose and insulin levels were considered as key endpoints for characterisation of this type 2 diabetes model which is widely used in interventional studies focusing on beta cell effects. To additionally investigate dynamics in the pancreatic endocrine cell pool we further assessed islet number and proliferation and apoptosis of beta cells in the 5-, 10-, and 24-week cohorts of animals. 

## Materials and Methods

### In vivo

#### Db/db mice

All animal experiments were conducted in accordance with internationally accepted principles for the care and use of laboratory animals. The study was approved by the Danish Committee for Animal Research and covered by an institutional licence issued to Zealand Pharma A/S (permit number: 2009/561-1633). The study included 72 male db/db (BKS.Cg-m +/+ Leprdb/J) mice, 5-6 weeks old at arrival, obtained from Charles River, Calco, Italy. Upon arrival, the mice were housed in groups of 4 in a light-, temperature-, and humidity-controlled room (12-hour light: 12-hour dark cycle, lights On/Off at 0600/1800 hour; 22±1°C; 50±10% relative humidity). All animals had free access to standard chow (Altromin 1324, Brogaarden A/S, Lynge, Denmark) and domestic quality tap water with added citric acid to achieve a pH ~ 3.6. Animals were randomised on arrival according to HbA_1c_ and fasted blood glucose levels in groups of 8 and terminated at 5, 6, 8, 10, 12, 14, 18, 24 and 34 weeks of age. 

#### Termination

Body weight was recorded the day before termination. Animals were fasted for 4 hours prior to termination with CO_2_ anaesthesia and subsequent cervical dislocation. Samples were collected for determination of terminal fasted blood glucose, HbA_1c_, insulin and C-peptide levels. Blood samplings and measurements were performed as previously described [25]. Subsequently, pancreata were removed *en bloc*, immersion-fixed in 4% formaldehyde (4% formaldehyde in 0.1M phosphate buffer; PBS pH 7.4) and stored at 4°C degrees until further processing.

### Pancreas immunohistochemistry

#### Embedding and sectioning

Dissected pancreata were processed as previously described [27]. Briefly, the pancreas was rolled into a cylinder, infiltrated with paraffin overnight and cut into four or five systematic uniform random tissue slabs with a razor blade fractionator. The slabs were embedded on their cut surface in one paraffin block. Subsequently, the blocks were trimmed before two 5 µm sections (300 µm apart) were cut from each block on a Microm HM340E (ThermoScientific, Walldorf, Germany) and arranged on one object slide representing in total a systematic uniform random sample of the whole pancreas. Two series were sampled from each animal: one for beta and non-beta cell immunohistochemistry and one for apoptosis. Two subsequent 4 µm sections (as reference and look-up sections) were cut and arranged on one object slide for quantification of islet number and cell proliferation. These sections were sampled at two levels (300 µm apart). For islet number estimation, the distance between the two neighbouring sections was increased to 12 µm. Based on a manual assessment of serial sections it was estimated that islets were not smaller than 15 µm and therefore islet numbers were estimated using non-serial sections to increase efficiency.

#### Immunohistochemistry

The paraffin-embedded sections were processed for double immunohistochemistry against insulin and an antibody cocktail against pancreatic polypeptide, somatostatin and glucagon as a measurement of beta and non-beta cells. The sections were immunostained manually using optimized staining protocols with diaminobenzidine (DAB) and DAB-nickel as chromogens as previously described [25]. The double labelling against Ki-67 and insulin as a measure of beta cell proliferation was performed using a similar approach. However, a rat anti-mouse Ki-67 antibody (DAKO M7249, Glostrup, Denmark) diluted 1:200 was used instead as a substitute for the non-beta cell antibody cocktail. This was followed by a secondary non-biotinylated rabbit anti-rat antibody (Vector Laboratories #AI-4001) diluted 1:200. The reaction was amplified for 30 min using a MACH2 goat anti-rabbit HRP polymer (BioCare Medical, Concord, CA 94520, USA, #RHRP 520H 040307). Islet apoptosis was visualized using a rabbit-anti-mouse caspase-3 antibody detecting the cleaved (activated) form of caspase-3 (1:50, Cell Signalling Technology, Danvers, MA #9661). 

#### Stereological estimation of pancreatic beta cell mass, number of islets, proliferation and apoptosis

Stereological estimations were performed using the newCAST system (Visiopharm, Hørsholm, Denmark) on virtual images obtained using an Aperio ScanScope® scanner with a 20x objective. Beta and non-beta cell mass estimations were performed by point-counting using a grid system with 12×12 =144 points at 20x magnification. All points hitting the structure of interest were counted. Pancreas volume was estimated using a 1-point grid per frame and, similarly, a 9-point grid system was used to correct for the presence of non-pancreatic elements in the dissected sample. The number of points hitting the structure of interest was converted into mass by taking the grid ratio into consideration [28]. Caspase 3 mass was estimated using a similar approach. 

To investigate the dynamics of beta cells, we examined proliferation by quantifying Ki-67 positive cells, and apoptosis by assessing caspase 3 positive cells in 5-, 10-, and 24-week-old animals. Quantitative estimates of the number of Ki-67 positive beta cells were performed by manual assessment of double labelled cells using the physical disector principle for pairing the reference and look-up section. A cell was counted only if it appeared on the reference and not the look-up section. The numerical density was estimated by counting cells within a reference volume defined by the area of an unbiased counting frame and the distance between the two neighbouring sections [29]. Lastly, the total number of Ki-67 positive cells was obtained by multiplying the numerical density with the total reference (beta cell) volume. 

Islet numbers were likewise estimated using the dissector principle where an islet was defined as a cluster of at least three endocrine cells as described by Bock et al [28]. The mean islet mass was calculated as the total volume of islets (beta + non-beta cell mass) divided by the total islet number. 

#### Statistics

Graphical presentations, calculations and statistical analyses were carried out using GraphPad software (GraphPad Prism version 5.04 for Windows, San Diego, California, USA). Statistical analysis was performed using a one-way analysis of variance (ANOVA) followed by Tukey's Multiple Comparison Test (P < 0.05 was considered significant). Linear correlations of beta cell mass to blood glucose were performed on young animals (5, 6, 8, 10, and 12 weeks of age) and in the older animals (14, 18, 24 and 34 weeks of age) to describe dynamics of beta cell mass in young versus older mice. Correlations were performed using the Spearman correlation. Results are presented as mean ± SEM.

## Results

### Development of type *2* diabetes in the db/db mice

**Figure 1:**
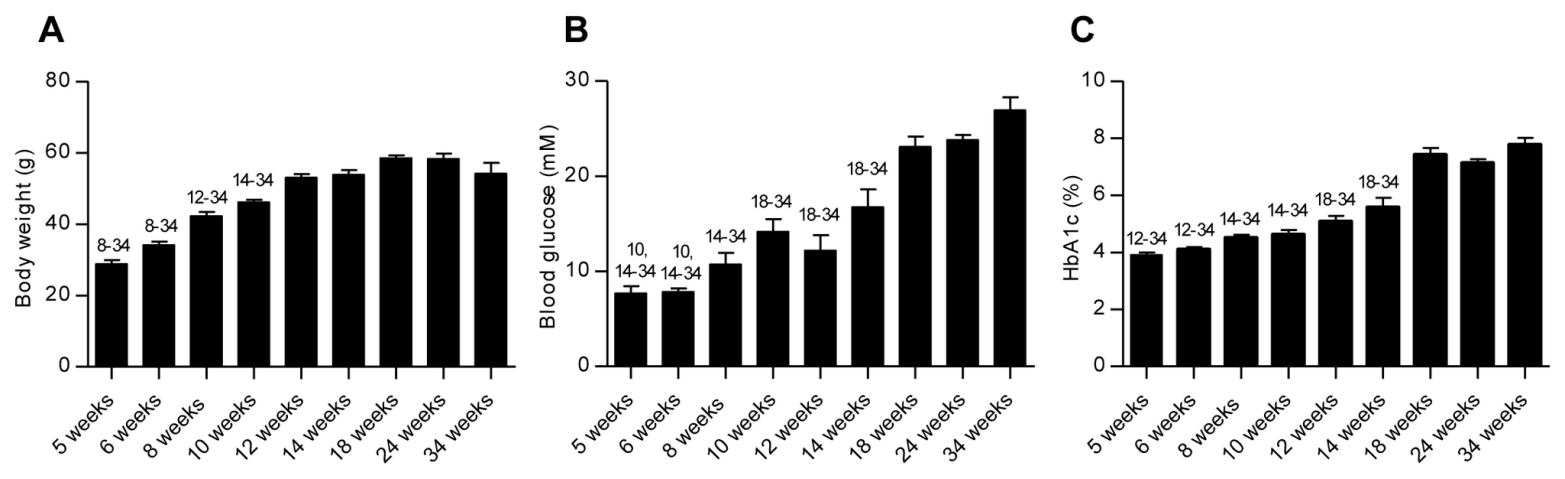
Development of T2D in the db/db mice. The progression of diabetes over time is shown as an increase in body weight (A), fasted blood glucose (B), and HbA_1c_ (C). Values were measured at termination in male db/db mice aged from 5 to 34 weeks. Statistically significant differences (P < 0.05) between time points are shown as numbers (corresponding to age in weeks) above the relevant bar graphs: Data are mean values with SEM and n = 8 in each group.

Body weight data from all groups are shown in Figure 1A. An increase in body weight was observed between the 5 and 12-week-old groups, after which a plateau was reached. The fasting blood glucose development is shown in Figure 1B. Starting at a mean fasted blood glucose value of 7.7 ± 0.8 mM in the 5-week-old group, blood glucose levels increased gradually to 26.9 ± 1.4 mM in the 34-week-old group. Hyperglycaemia was reached in mice at 8 weeks of age with a mean fasted blood glucose value of 10.8 ± 1.2 mM. HbA_1c_ levels (Figure 1C) gradually increased from 3.9 ± 0.1% in 5-week-old mice to 7.8 ± 0.2 % in the 34-week-olds. 

**Figure 2:**
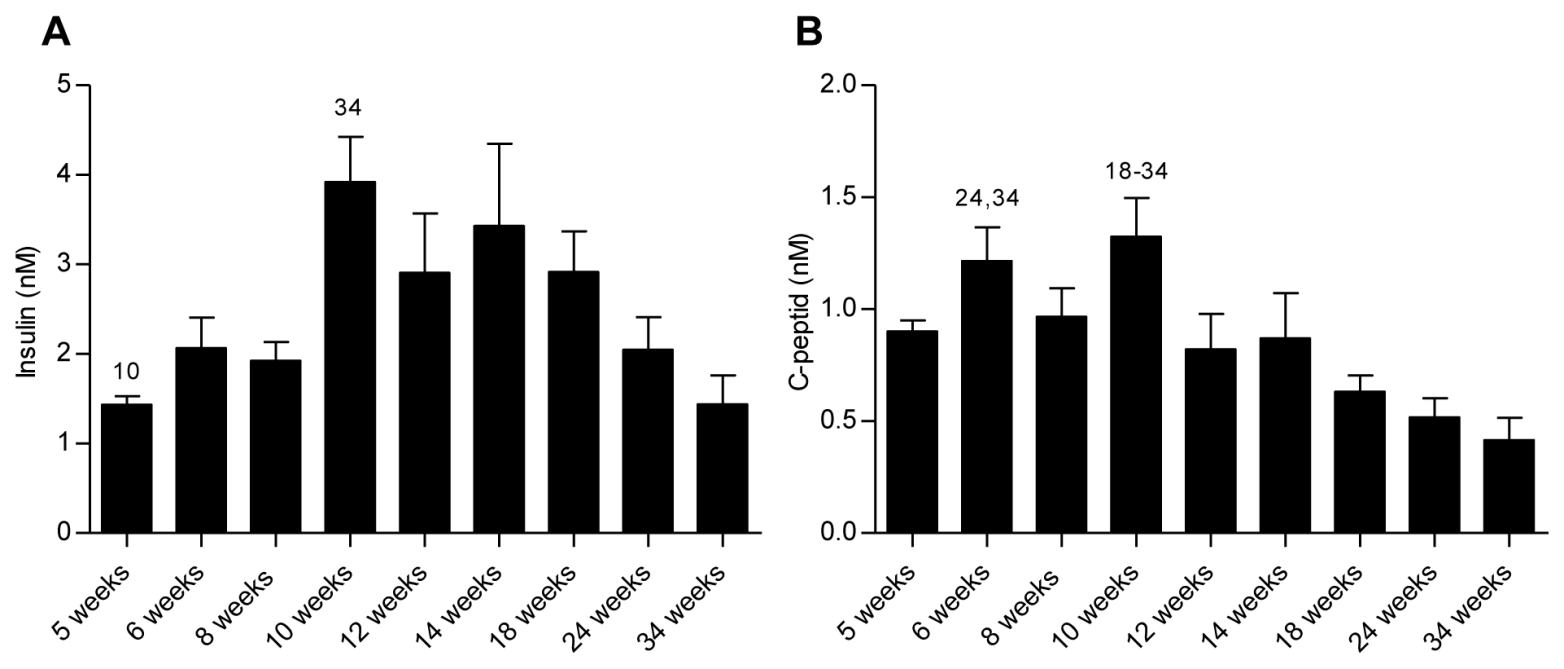
Fasted plasma insulin and C-peptide. Insulin (A) and C-peptide (B) values were measured at termination. Statistically significant differences (P < 0.05) between time points are shown as numbers (corresponding to age in weeks) above the relevant bar graphs. Data are mean values with SEM and n = 8 in each age group.

Plasma insulin and C-peptide levels are shown in Figure 2. The plasma insulin concentrations (2A) increased significantly (P < 0.05) from 1.43 ± 0.09 nM at 5 weeks of age to a peak value of 3.92 ± 0.50 nM at 10 weeks, whereafter insulin levels declined to the lowest level of 1.44 ± 0.32 nM at 34 weeks of age. A similar pattern was observed for plasma C-peptide concentrations with a peak value at 10 weeks of age (1.33 ± 0.17 nM) followed by a subsequent decline leading to significantly lower values at 18 (0.63 ± 0.0.07 nM, P < 0.05), 24 (0.52 ± 0.09 nM, P < 0.01), and 34 (0.42 ± 0.10 ng/ml P < 0.001) weeks of age.

### Age-dependent pancreatic islet morphology

**Figure 3:**
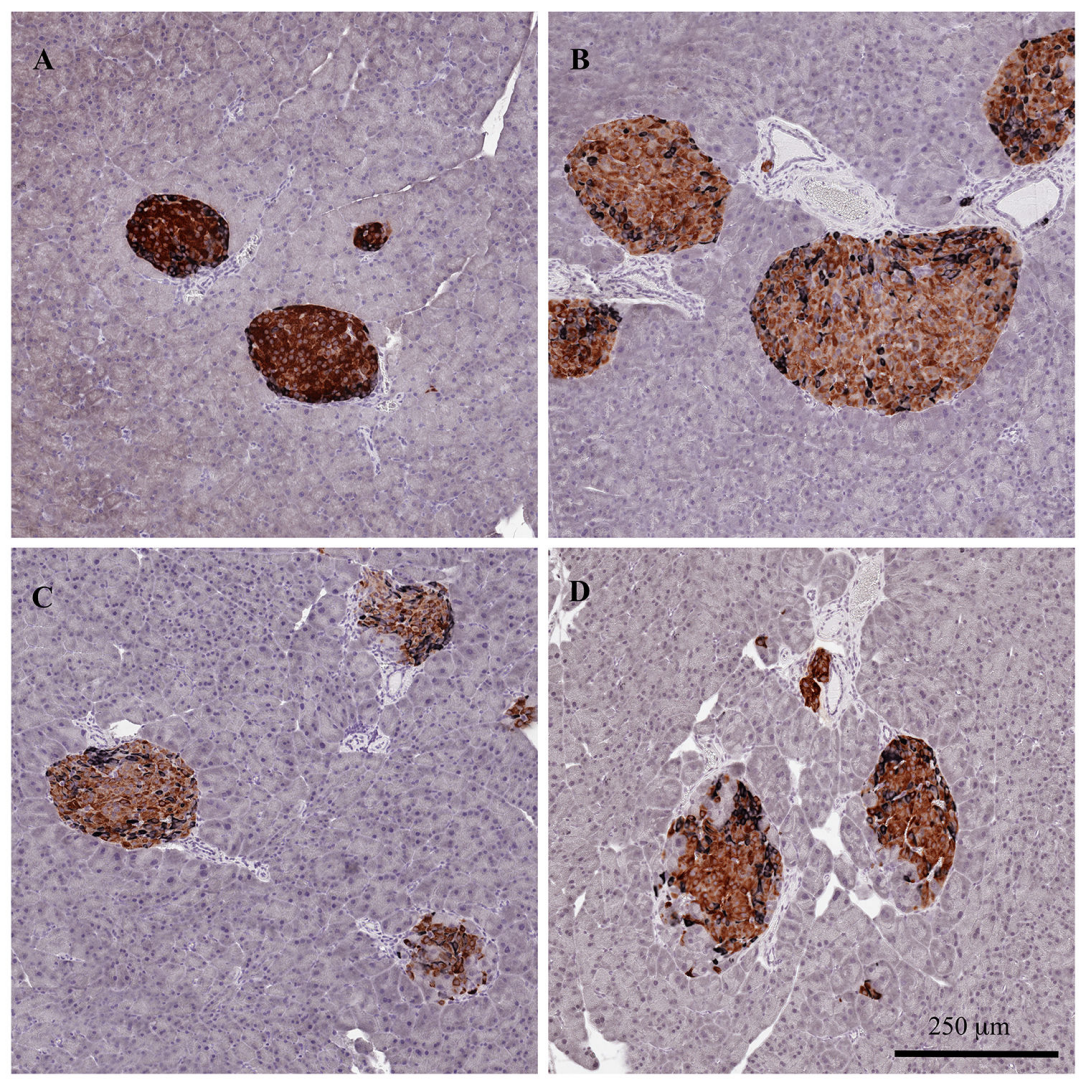
Age-dependent pancreatic islet morphology in db/db mice. Representative images of age-dependent islet morphology at 5 (A), 12 (B), 24 (C), and 34 (D) weeks of age. Sections were immunohistochemically stained for insulin and an antibody cocktail against pancreatic polypeptide, somatostatin and glucagon as a measurement of beta (brown) and non-beta cells (black).

Representative images of age-dependent islet morphology at 5, 12, 24 and 34 weeks of age are shown in Figure 3. At 5 weeks of age the islets had a regular shape with beta cells located in the centre of the islet and the non-beta cells in the periphery. At 12 weeks of age the islets appeared to have increased in size, while their architecture was still intact. 

In contrast, a marked deterioration in normal islet structure was observed in older animals aged 24 and 34 weeks. The immunohistochemical staining pattern for beta and non-beta cells was irregular with non-beta cells scattered within the interior of the islets. The islets appeared smaller than in animals at 12 weeks of age and infiltrating acinar cells were observed within the islets. 

### Stereological estimation of beta cell, non-beta cell and islet mass

**Figure 4:**
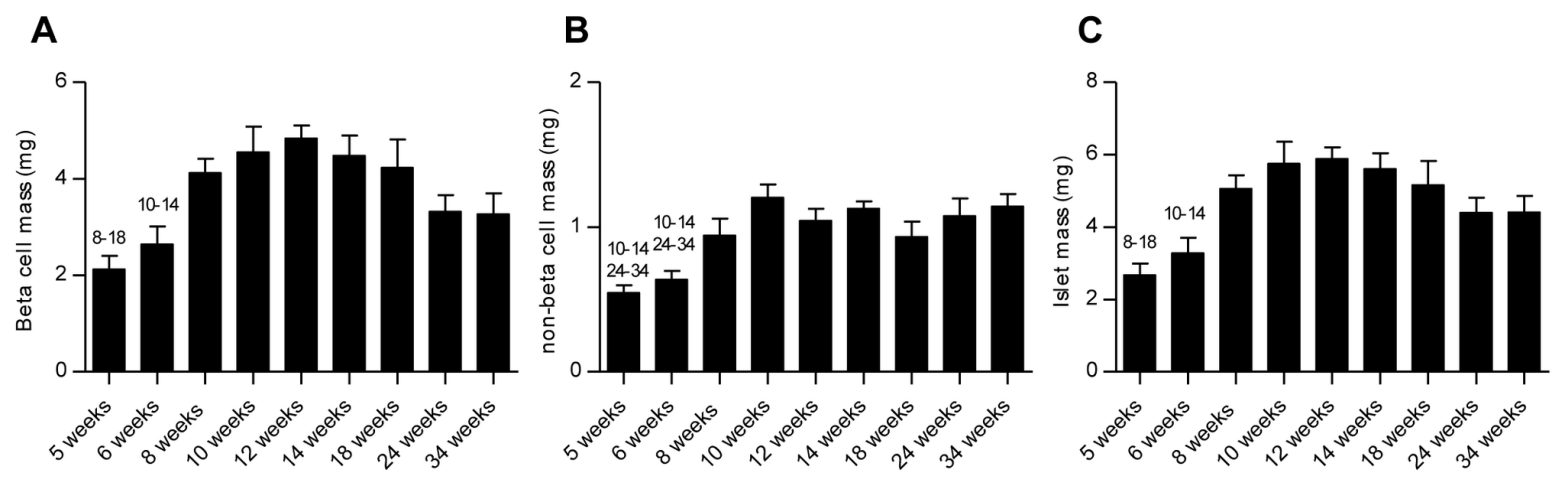
Stereological estimation of beta cell, non-beta cell and islet mass in male db/db mice at different ages. Total pancreatic beta cell mass (A) and islet mass (C) increased in the young animals and then gradually decreased in the older animals. Following an initial increase no apparent changes were observed in non-beta cell mass (B). Statistically significant differences (P < 0.05) between time points are shown as numbers (corresponding to age in weeks) above the relevant bar graphs. Data are mean values with SEM and n = 8 in each age group.

The stereological analysis of beta cell mass is shown in Figure 4A. Starting at an initial mass of 2.1 ± 0.3 mg at 5 weeks of age a significant increase was observed across the groups up to mice aged 12 weeks with a peak value of 4.84 ± 0.26 mg (P < 0.05). From 14 weeks of age and onwards the mean pancreatic beta cell mass declined slightly to a mean of 3.3 ± 0.4 mg in the 34-week-old group. A similar pattern was observed for islet mass (Figure 4C). The non-beta cell mass is depicted in Figure 4B. Following an initial significant increase from 0.5 ± 0.1 mg at week 6 to 1.2 ± 0.1 at 10 weeks of age, a plateau was reached with no apparent changes in non-beta cell mass from week 12 to week 34. 

### Relationship between pancreatic beta cell mass and fasted plasma glucoses levels

**Figure 5:**
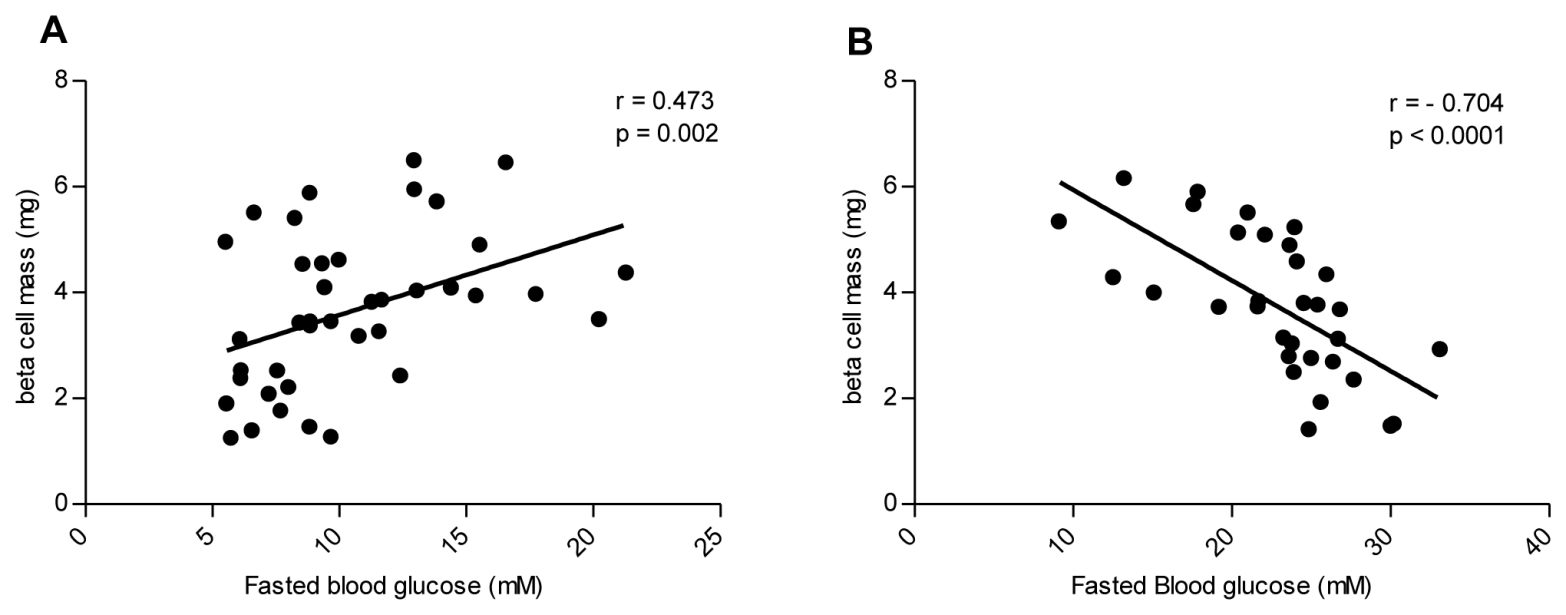
Relationship between pancreatic beta cell mass and fasted terminal blood glucose values. A correlation between increasing beta cell mass and increasing blood glucose values is seen in animals of 5-12 weeks of age (A). A decrease in beta cell mass was correlated with an increase in blood glucose values in animals of 14-34 weeks of age (B).

The beta cell mass as a function of terminal fasted blood glucose values from the 5-12 week groups and the 14-34 week groups are depicted in Figure 5A and 5B, respectively. In the young animals (5-12 weeks), the age-dependent increase in plasma blood glucose levels was significantly correlated with an increase in beta cell mass (r = 0.473, P = 0.002). Conversely, a decrease in beta cell mass in the older animals (aged 14-34 weeks) was significantly correlated with an increase in blood glucose levels (r = -0.704, P < 0.0001). 

### Islet number and mean islet mass

**Figure 6:**
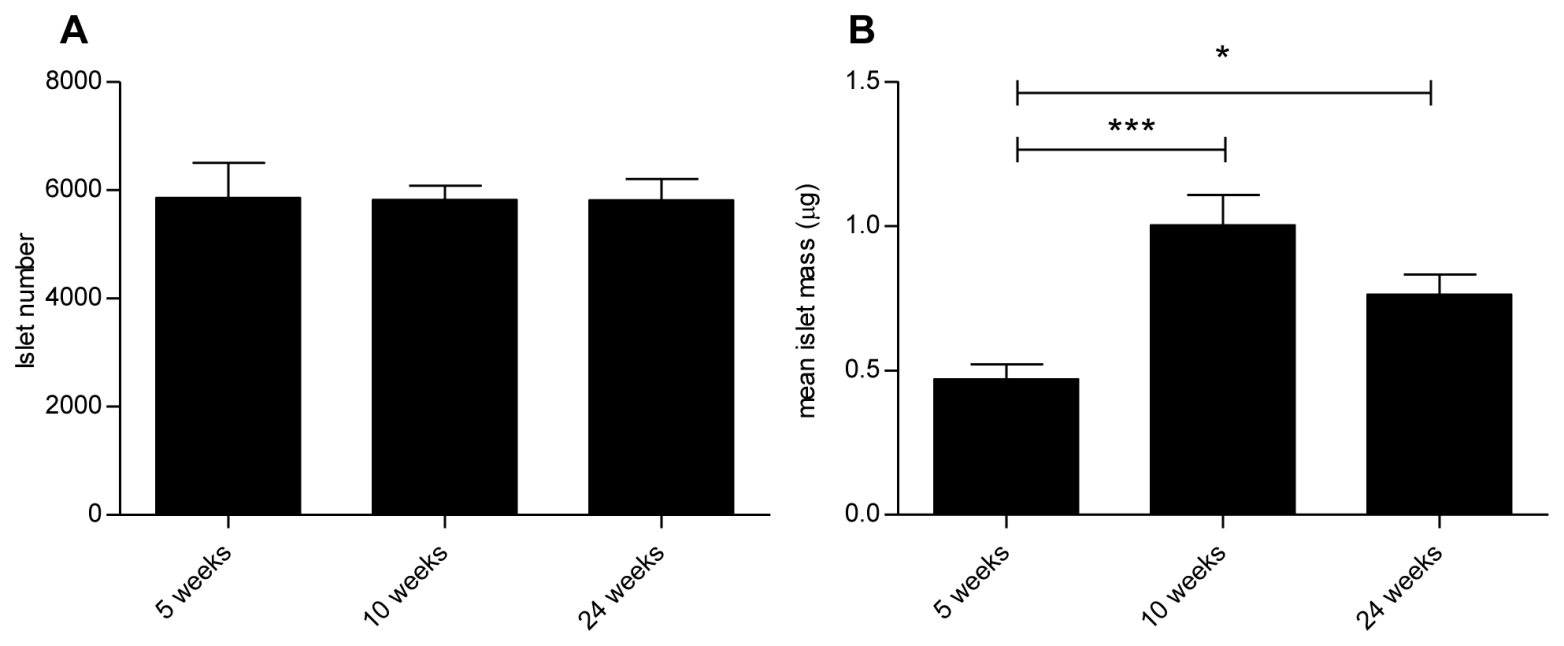
Stereological estimation of islet numbers and mean islet mass. No significant differences were observed in islet numbers (A) between the age groups, whereas significant differences were observed in mean islet mass (B). Data are mean values with SEM and n = 8 in each age group * = p < 0.05, *** = p <0.001.

The stereological estimation of the total number of islets and mean islet mass in 5-, 10- and 24-week-old db/db mice is shown in Figure 6A and 6B, respectively. The number of islets was similar between the 5-, 10- and 24-week-old mice (5859 ± 648, 5820 ± 266 and 5816 ± 393, respectively). In contrast, a significant increase in mean islet mass was evident in the 10 (1.0 ± 0.1 μg) and 24- week-old (0.8 ± 0.1 μg) mice compared with 5-week-old (0.5 ± 0.1 μg) animals, demonstrating that the increase in beta cell mass was driven by an increase in islet mass and not islet number. 

### Beta cell proliferation and apoptosis

**Figure 7:**
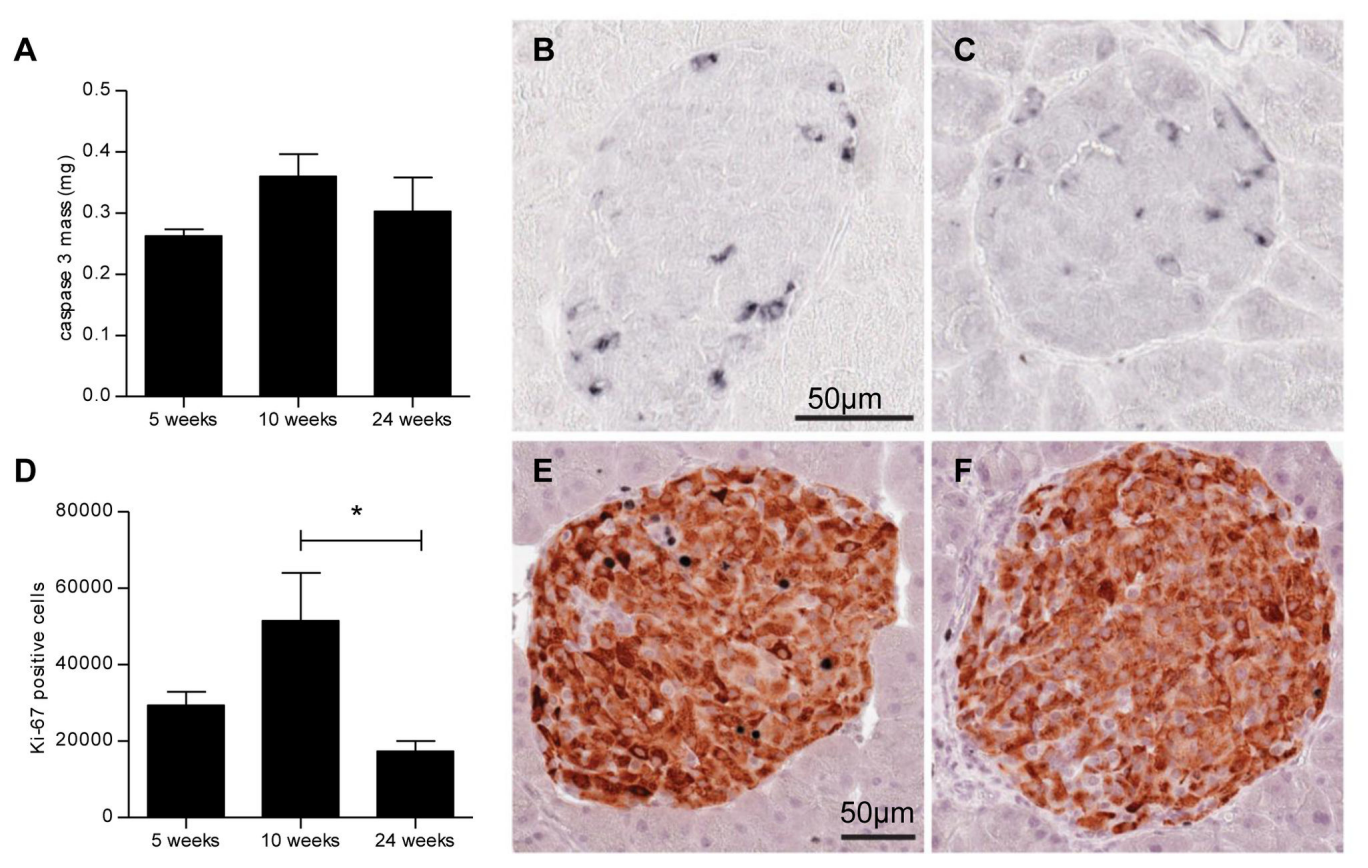
Beta cell proliferation and apoptosis. Stereological estimation of mean caspase 3 mass (A) and the number of proliferating Ki-67 immunoreactive beta cells (D). Immunohistochemical staining for cleaved caspase 3 from animals aged 10 (B) and 24 (C) weeks and for insulin (brown) and Ki-67 positive nuclei (black) from animals aged 10 (E) and 24 (F) weeks. Data are mean values with SEM and n = 8 in each age group, * = p < 0.05.

The number of proliferating Ki-67 immunoreactive beta cells was significantly higher in the 10- week-old mice than in the 24-week-old mice (Figure 7). No apparent differences were observed between the 5-, 10- and 24-week-old mice in the mass of caspase 3 immunoreactive apoptotic islet cells. 

## Discussion

The present study investigated changes in beta cell dynamics and blood glucose levels in groups of young to old male C57BLKS/J db/db mice in order to characterise changes in pancreatic beta cell mass during the development of glucose intolerance in this widely used type 2 diabetes animal model. The study relates to beta cell dynamics in male db/db mice only. In this respect it is important to note that progression of diabetes can vary between sexes [30,31]. Thus, the current results may not readily be used to describe beta cell dynamics in female db/db mice. Our data suggest that an increase in beta cell mass was accompanied by increasing insulin and blood glucose levels in younger mice up to 12 weeks of age. After reaching a peak value in 12-week-old mice, the beta cell mass started to decline while blood glucose levels increased and insulin levels decreased. Although the db/db mouse has been widely used in studies addressing compound-induced changes in beta cell mass [23-26] the age-related beta cell dynamics in untreated male db/db mice during diabetes development and progression have not been investigated in detail.

The physiological characteristics of the development of diabetes in the db/db mice have been investigated in a number of studies [17,18,32]. In alignment with these reports we demonstrated that the db/db mice become obese and develop diabetes from approximately 8 weeks of age as shown by clear differences in their plasma glucose levels. In the present study we coupled changes in plasma parameters of glucose and insulin with a thorough examination of beta cell mass. Previously, changes in beta cell mass in 7-, 8-, 10- and 12-week-old C57BLKS/J db/db mice were investigated by Puff et al. [22]. In contrast to our study, Puff et al observed a slight decrease in beta cell mass between 7 and 12 weeks coupled with a decrease in blood glucose values at week 12. The reason for this discrepancy between studies is not known, but it might be related to the different sampling technique used and the fact that Puff et al. only analysed one section per animal in 3-6 subjects, which might result in a biased beta cell mass estimation. Unfortunately, Puff et al. did not include mice older than 12 weeks, thereby preventing an investigation of the beta cell mass during late progression of diabetes. Kanda et al. [33] and Kawasaki et al. [23] quantified beta cell mass in 8 and 12, and 12 and 18-week-old animals, respectively. In agreement with our study Kanda et al. described an increase in beta cell mass from 8 to 12 weeks of age [33]. Kawasaki et al. [23] reported a decrease in beta cell mass from week 12 to week 18, further supporting our results of age-dependent changes in the pancreatic endocrine cell pool.

It has previously been suggested that, at least in rodents, beta cells to some degree can compensate for an increased metabolic load and insulin demand by increasing their beta cell mass and function in an attempt to maintain euglycaemia [9,10,34,35]. Our findings are in agreement with this, as we found that beta cell expansion was correlated with increasing blood glucose levels in the young animals. The increase in beta cell mass in the young animals occurred concurrently with an increase in insulin and C-peptide levels. Thus supporting the hypothesis that the young animals could increase pancreatic beta cell mass and insulin secretion to overcome hyperglycaemia and insulin resistance as previously described [9,10,34,35]. However, the ability to expand beta cell mass was not unlimited. At 12 weeks of age the maximum capacity was reached and was followed by an inversely correlated decline in beta cell mass and increase in blood glucose values, accompanied by decreasing insulin concentrations [17,19,21,23,33].

In order to investigate the dynamics of beta cells, we examined proliferation by quantifying Ki-67 positive cells and apoptosis by assessing cleaved caspase 3 positive cells at three different time points. The investigation included the pre-diabetic (aged 5 weeks), early diabetic (10 weeks) and late stage diabetic mice (24 weeks old). In line with others, we reported an increased proliferation of beta cells in the 10-week-old animals [21,36], and decreased proliferation in the older animals [21,37], indicating that changes in beta cell mass are related to the fine balance between apoptosis and proliferation [9]. The exact molecular mechanism leading to functional adaptation of beta cells is unknown. However, studies suggest that increased sensitivity to free fatty acids and glucose may cause beta cell mass to expand [9,34,35,38,39]. Although increased glucose and lipid levels might trigger an initial beta cell mass expansion, extremely high levels have also been shown to be toxic, and several lines of evidence suggest that glucolipotoxicity plays a crucial role in beta cell apoptosis [40-44]. Prolonged exposure to high glucose levels has been shown to be toxic to beta cells *in vitro* [43,44] and prolonged exposure to fatty acids has been reported to increase beta cell apoptosis [44,45]. Moreover, high levels of glucose and free fatty acids in combination appear to induce apoptosis to an even greater extent than glucose and free fatty acids alone [40]. In contrast to the above reports, we did not observe a statistically significant increase in apoptosis. This discrepancy may be methodology (i.e. *in vitro* vs. *in vivo* settings) or biologically driven. In contrast to our study, Puff et al observed an increase in apoptosis related to increasing age in the db/db mouse [22]. However the inherent difficulties in apoptosis quantification *in vivo*, due to the rapid clearance of dying cells, as found in this report and in the Puff et al. study, might account for this variation [46]. Additionally, we cannot exclude the possibility that apoptosis might have occurred at an earlier stage not detected by our analysis. The molecular mechanism leading to the decrease in beta cell mass was not further pursued in this study. However, accumulating evidence indicates that changes in islet associated transcription factors, which are important for the beta cell differentiation, such as pancreatic duodenal homebox-1 (PDX-1), Nkx6.1, beta-cell E-box trans-activator 2 (B2/NeuroD) and paired box gene 6 (Pax6), have been shown to be reduced with age in islets of db/db mouse [33,47]. Increase in inflammatory cytokines like interleukin-1 beta (IL-1β) have also been suggested to contribute to beta cell apoptosis [43,48]. Additionally, dysregulation of the mammalian target of rapamycin (mTOR) pathway has shown to play an important role in the regulation of beta cell mass [49]. Furthermore, mitochondrial dysfunction caused by reactive oxygen species (ROS) as a consequence of increased metabolism of glucose and free fatty acids is a central contributor to beta-cell apoptosis [41,50,51]. Therefore, collectively, it appears that the hyperglycemic and hypertriglyceridemic features could likely contribute to the beta cell mass decline in old db/db mice [17-19]. 

To obtain a better understanding of the changes in beta cell dynamics we have finally aimed to elucidate whether the beta cell mass expansion was caused by increased islet numbers (hyperplasia) or increased islet mass (hypertrophy). Using a stereological approach we demonstrated that age-dependent expansion of beta cell mass is driven by an increase in mean islet mass rather than formation of new islets. This is in line with previous reports from the ob/ob mice [28], reporting that islet neogenesis does not occur spontaneously and that cells within the existing islets are the most important source of expansion of beta cell mass. Similarly, Dor et al. [52] reported that the primary source of new beta cells was proliferation rather than stem cell formation. 

Potential anti-diabetic treatments preventing beta cell decline and possibly targeting beta cell regeneration are currently gaining increased interest [53,54]. However, to evaluate and predict a proliferative effect of pharmacological treatment on beta cells, a reliable animal model is needed. In this study, we suggest but do not demonstrate that beta cell mass in the db/db mice responds in a dynamic way to compensate for increased insulin demand. The relationship between beta cell mass, blood glucose and insulin levels was previously investigated by our group in the male ZDF rat [27]. However, beta cell dynamics in the ZDF rat model seemed to be more dramatic, with a rapid expansion and subsequent marked decline in beta cell mass within a short 10-week period. No correlation between beta cell mass and beta cell functionality was observed in the ZDF rat. In contrast, in the db/db mouse a relationship between beta cell mass and beta cell function was observed, highlighting the db/db mouse as a good model to evaluate potential proliferative effects of various interventions (pharmacological or non-pharmacological) on beta cell mass and function. 

Based on the analysis of the 5-, 10-, and 24-week age groups we hypothesized that in the db/db mouse, beta cell mass expansion in younger animals was a consequence of increased proliferation of the cells, and the decrease in beta cell mass in older mice was related to a decrease in the beta cell proliferation. Several studies have reported an increase in beta cell mass in obese non-diabetic human subjects [6,7,55,56]. However, those studies did not show any increase in beta cell replication, suggesting that the adaptive increase in beta cell mass in humans and rodents may be the result of different mechanisms. The inherent limitation of human studies lies in their attempt to capture dynamic changes with static criteria. Beta cell proliferation measurements are taken at single time points and thus dynamic changes cannot be recorded and accounted for [57]. Rahier et al. indicated that beta cell mass in T2D patients declines over time, starting from the time of diagnosis [8], as we found in older db/db mice. Several studies based on human autopsies have also shown decreased beta cell mass in T2D patients with increased apoptosis [6,7,55,58], suggesting a selective beta cell loss in the pathogenesis of T2D. Although we do not claim a direct link between the db/db mouse model and humans, we believe that such data may give a useful insight into the dynamics of decreased beta cell mass in T2D patients [57].

In conclusion, we provide a detailed characterisation of the age-dependent beta cell dynamics during the development of glucose intolerance in the male C57BLKS/J db/db mice. Our findings suggest that age-dependent beta cell dynamics in male db/db mice can be coupled to alterations in insulin/glucose homeostasis. In young db/db mice, high beta cell proliferation is seen, resulting in an increase in beta cell mass, which is believed to compensate for an increased insulin demand. The subsequent decrease in beta cell mass in older animals is characterised by a low beta cell proliferation. We further demonstrated that beta cell expansion is caused by an increase in the mean islet mass and not islet number. Utilisation of a non-biased stereology approach increases the value of our findings as a reference for future beta cell studies in this T2D animal model. 

### Study limitations

The pancreatic beta and non-beta cells were identified from their insulin, glucagon, somatostatin, and pancreatic polypeptide immunoreactivity, and thus the functional state of the beta and non-beta cells might affect the reactivity of the antibodies. In addition, quantification of apoptosis was difficult due to the rapid clearance of dying cells [6,46]. Therefore, we were not able to perform a reliable double stain with insulin and caspase 3 immunoreactivity. Thus, apoptosis was quantified in the whole islet whereas Ki-67 was quantified in beta cells only. 
